# London and Counties Medical Protection Society : Notice

**Published:** 1909-07-10

**Authors:** Hugh Woods, A. G. R. Foulerton

**Affiliations:** General Secretary; Financial Secretary


### LONDON AND COUNTIES MEDICAL PROTECTION SOCIETY: NOTICE.

An extraordinary general meeting of the members of the London and Counties Medical Protection Society, Limited, is announced for Wednesday next, July 14, at 4 p.m., at the office of the Society, 31 Craven Street, Strand, London, W.C., when the following special resolution, which was carried by the requisite majority at an extraordinary general meeting on June 23, 1909, will be submitted for confirmation: That the Articles of Association of the Society be altered in manner following, viz.:—

To alter Articles (2), (3), (5), and (13) respectively so as to read thus:—(2) "Any registered medical or dental practitioner may, if accepted by the Council, become a member of the Society." (3) "Each candidate for membership shall sign and deliver to one of the Secretaries an application in such form as may be approved by the Council." (Omitting the words of the form at present included in the article.) (5) "The Council may, by giving 14 days' notice to any member whose conduct or membership is in the opinion of the Council detrimental to the Society, determine his membership, and thereupon he shall cease to be a member, but shall nevertheless pay all subscriptions and calls in arrear." (13) "The Council shall consist of a President, Vice-Presidents, one Treasurer, one General Secretary, one Financial Secretary, and 12 additional members of Council, who shall all be elected annually by the Society at its annual meetings, together with the Presidents of all districts recognised by the Council, who shall be *ex-officio* members of the Council. The quorum for a Council meeting shall be three."

Dated June 30, 1909.

Hugh Woods, *General Secretary.*
A. G. R. Foulerton, *Financial Secretary.*

